# 523 Reduced Incidence of Fractures After Treatment with Oxandrolone in Burn Patients

**DOI:** 10.1093/jbcr/irac012.154

**Published:** 2022-03-23

**Authors:** Elliot Walters, Kayleen Whitley, Steven E Wolf

**Affiliations:** University of Texas Medical Branch, Dickinson, Texas; Galveston, Texas; University of Texas Medical Branch at Galveston, Galveston, Texas

## Abstract

**Introduction:**

Bone density loss is a significant and well documented complication after major burns. Oxandrolone and bisphosphonates have both been used successfully to mitigate this outcome. Studies show these agents reduce both short-term and long-term bone loss, but no studies have examined the long-term clinical outcomes of these agents. This study investigates long-term outcomes of treatment with oxandrolone and bisphosphonates in burn patients.

**Methods:**

We examined a deidentified database of electronic medical records across 55 healthcare organizations including over 75 million patients. ICD 10 codes were used to identify patients with thermal or chemical burns from January 1, 2010 to December 31, 2020. We included patients who received their first dose of oxandrolone or bisphosphonate within one month of injury. Propensity score matching was used to balance patient cohorts. ICD 10 and CPT codes were used to evaluate outcomes.

**Results:**

We identified 280,367 patients with burn injuries during the study time period. Of these, 903 (0.32%) received at least one dose of oxandrolone and 307 (0.11%) received at least one dose of a bisphosphonate medication within 1 month of injury. Mortality was higher among matched patients receiving oxandrolone (OR: 3.146, CI: 2.224, 4.449) or a bisphosphonate (OR 3.027, CI: 1.8, 5.092). Fracture at any site and fracture of long bones were significantly lower among matched patients who received oxandrolone (OR: 0.704 CI: 0.542, 0.914, OR: 0.689, CI: 0.51, 0.931; respectively) compared to those who did not. No reduction of fractures was seen among patients who received bisphosphonates (p >0.05). Among patients receiving oxandrolone acute kidney failure was increased (OR: 1.941, CI: 1.454, 2.592) compared to those not receiving the medication but chronic kidney failure was reduced (OR: 0.513, CI: 0.351, 0.749). There was no increase in acute or chronic kidney failure among patients receiving a bisphosphonate (p >0.05). Liver injury was not increased among patients receiving either medication (p >0.05).

**Conclusions:**

Oxandrolone and bisphosphonate medications have been well studied and shown to decrease bone density loss after burn injury. Fractures of all bones and specifically long bones were reduced in patients receiving oxandrolone, suggesting that decreased bone catabolism during the acute recovery period may provide long-term injury protection. While we do see an increase in mortality with both of these medications, there is no we do not see any increase in liver failure or chronic kidney failure suggesting that factors unrelated to the administration of these medications are driving the increased mortality and may be related to selection bias. This is the first study to show that oxandrolone decreases the incidence of fractures after burn injury.

